# Supplementary material for: Screening for antibacterial and cytotoxic activities of Sri Lankan marine sponges through microfractionation: Isolation of bromopyrrole alkaloids from Stylissa massa
Source: PLoS One. 2024 Jan 8;19(1):e0296404. doi: 10.1371/journal.pone.0296404 (PMC10773956; doi:10.1371/journal.pone.0296404)
Supplement: S1 File — Includes images of the sponge species investigated in the current study, map of sponge sample collection sites in Sri Lanka, results of the agar disc diffusion assay, microfractionation exemplified by the isolation and identification of bromopyrrole alkaloids from Stylissa massa, a summary of sponge extracts that gave no antibacterial activity in the disc diffusion and microdilution assays and a literature review of antibacterial and cytotoxic compounds identified in previous reports for the same sponge species as in the current study. (DOCX) [file pone.0296404.s001.docx]

***Screening for antibacterial and cytotoxic activities of Sri Lankan marine sponges through microfractionation: Isolation of bromopyrrole alkaloids from Stylissa massa***

Lakmini Kosgahakumbura^1,2 ¶^, Jayani Gamage^1,2 ¶^, Luke P. Robertson^2^, Taj Muhammad^2^, Björn Hellman^3^, Ulf Göransson^2^, Prabath Jayasinghe^4^, Chamari Hettiarachchi^1^, Paco Cárdenas^2^, Sunithi Gunasekera^2*^

^1^ Department of Chemistry, University of Colombo, Kumaratunga Munidasa Mawatha, Colombo 03, Sri Lanka

^2^ Pharmacognosy, Department of Pharmaceutical Biosciences, Biomedical Centre, Uppsala, Sweden

^3^ Drug Safety and Toxicology, Department of Pharmaceutical Biosciences, Biomedical Centre, Uppsala, Sweden

^4^ Marine Biological Resources Division, National Aquatic Resources Research and Development Agency (NARA), Crow Island, Colombo 15, Sri Lanka

*****Corresponding author

Email: [sunithi.gunasekera@farmbio.uu.se](mailto:sunithi.gunasekera@farmbio.uu.se) (SG)

^¶^ These authors equally contributed to this work

Short title – Bioassay guided microfractionation of Sri Lankan sponges

**Table of contents**

| **Item** | **Page number** |
| --- | --- |
| **S1 Fig.** Illustrations of the 30 species collected from the 16 locations around Sri Lanka | 3 |
| **S2 Fig.** Map of the collection sites | 5 |
| **S3 Fig.** Agar disc diffusion assay | 6 |
| **S4 Fig.** Isolation and identification of bromopyrrole alkaloids from *Stylissa massa* 2 | 7 |
| **S1 Table.** Extracts with no antibacterial activity in the disc diffusion and micro dilution assays | 8 |
| **S2 Table.** Systematic literature review | 9 |
| References | 15 |


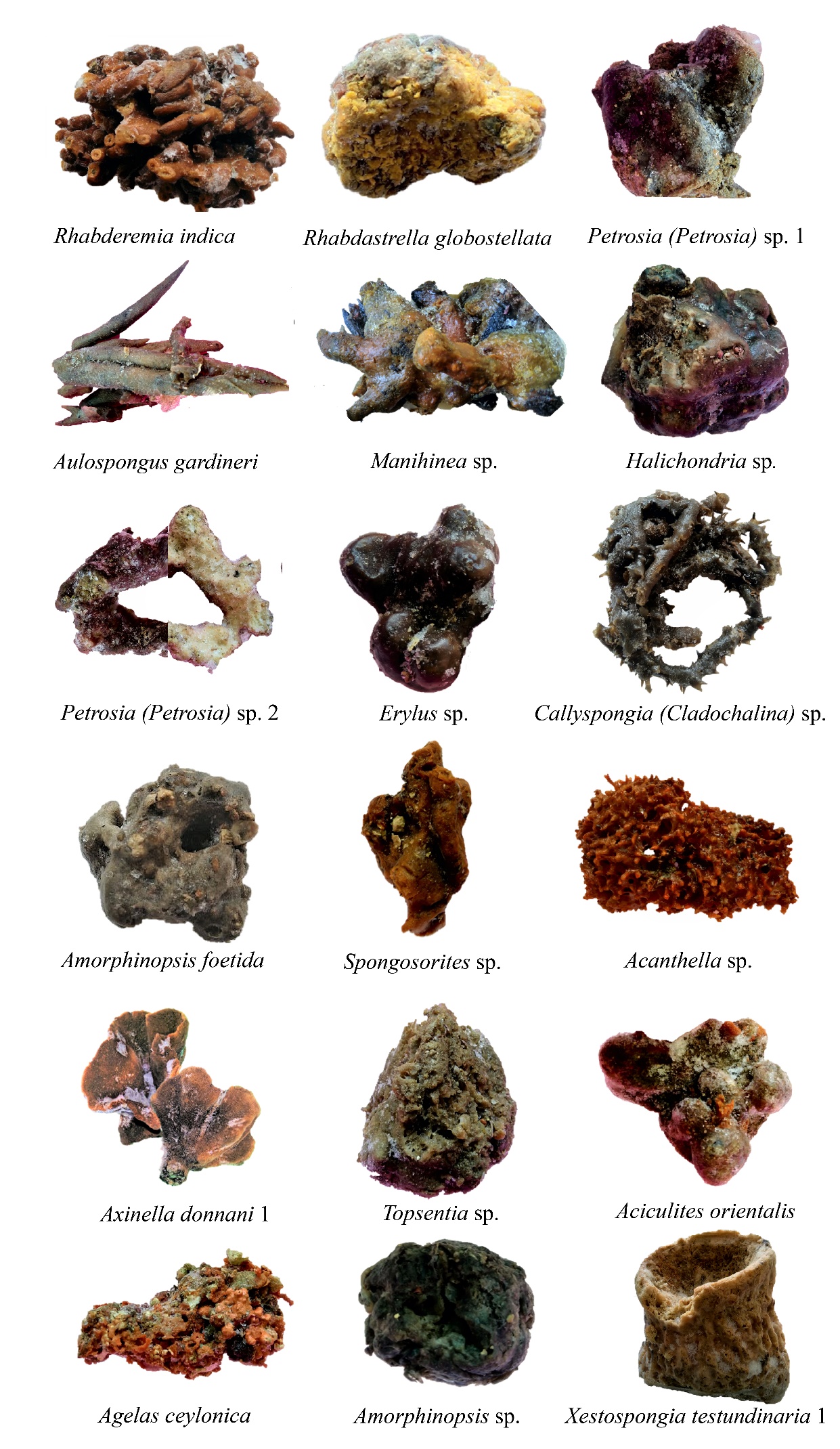


**
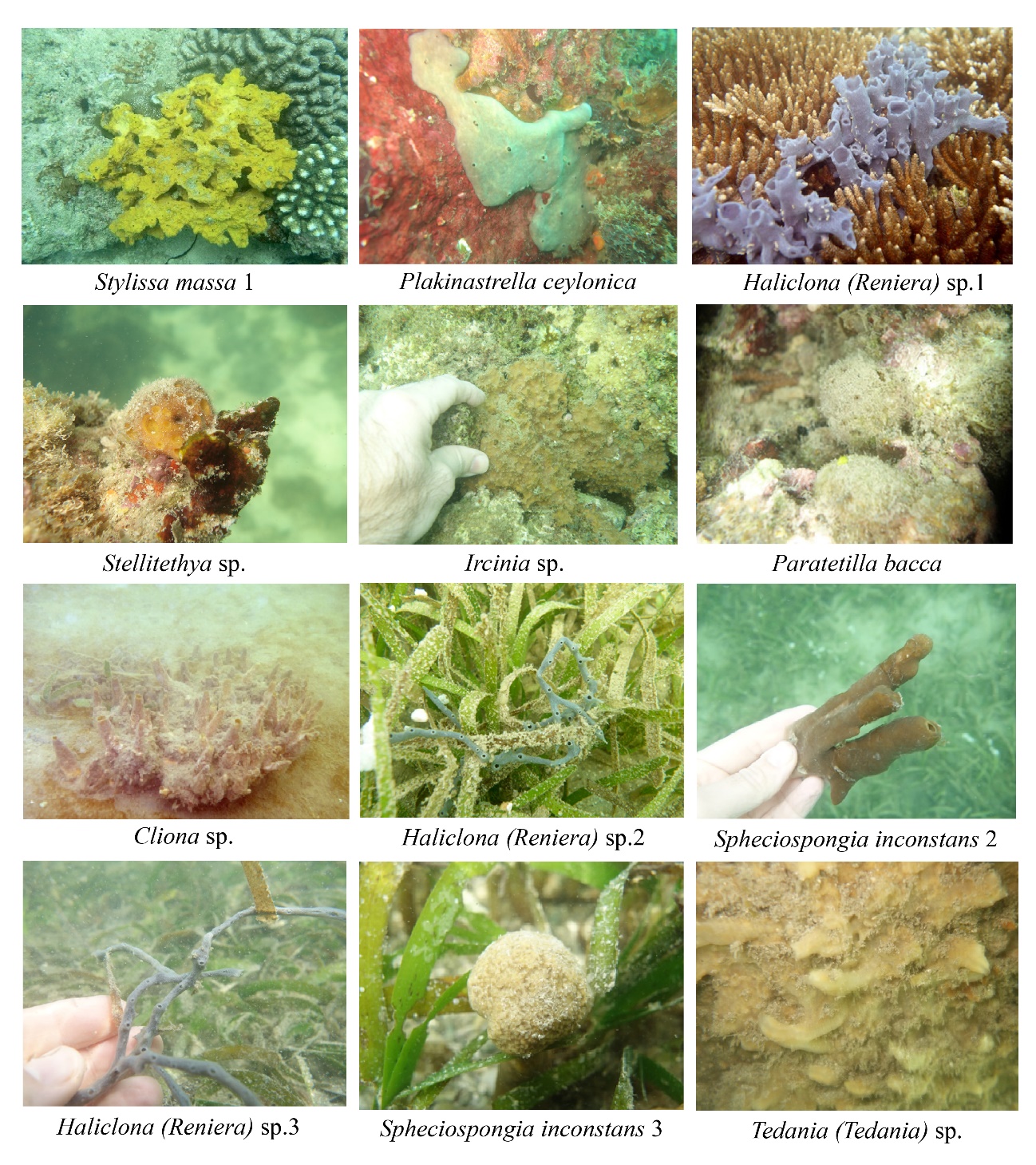
S1 Fig. Illustrations of the 30 species collected from the 16 locations around Sri Lanka.**


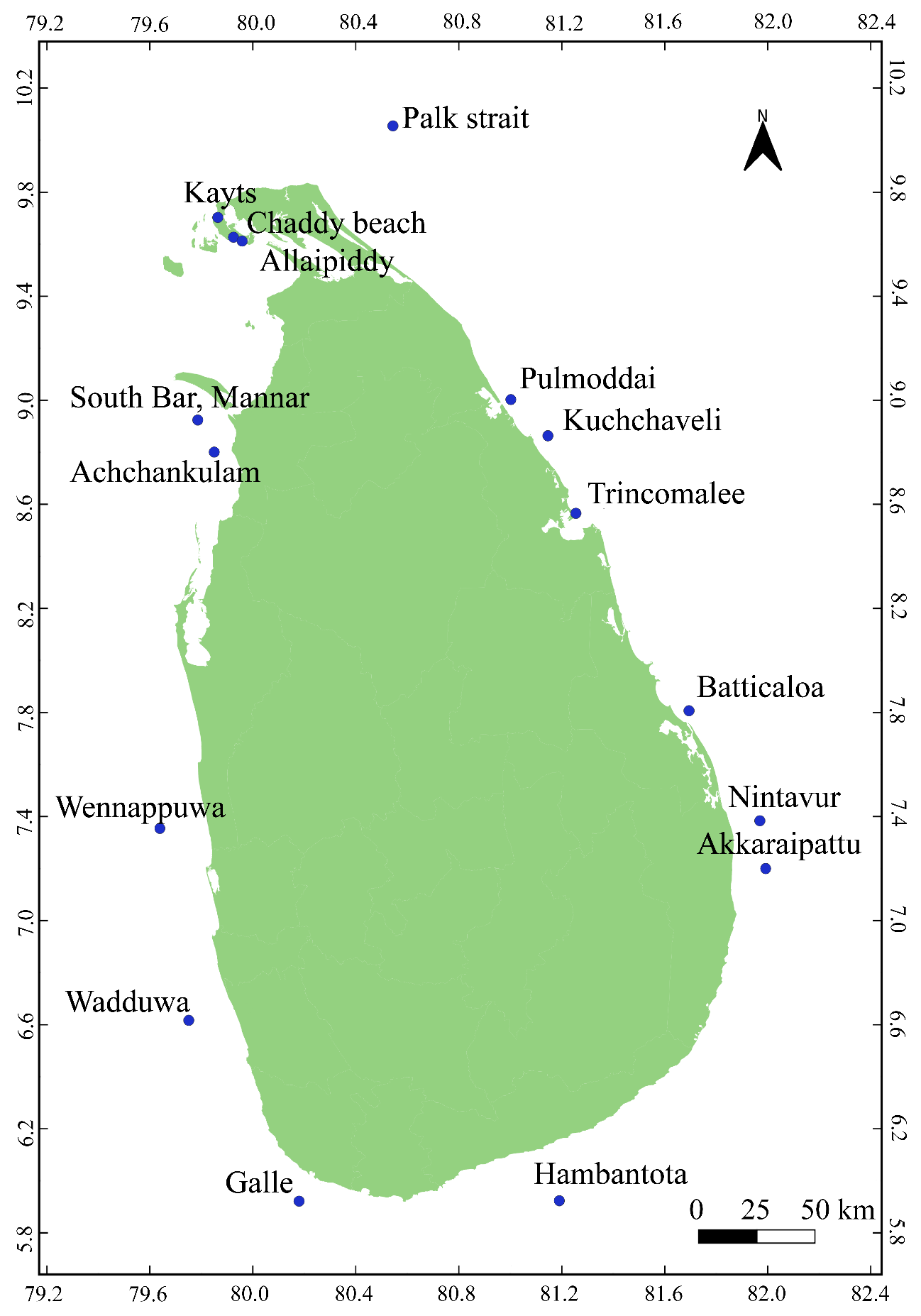


**S2 Fig. Map of the collection sites**. Specimens were collected from 16 locations around the coastline of Sri Lanka, from <1 to 80 m depth, by scuba-diving, snorkelling, and bottom trawling (cf. Table 1 for a list of the stations).


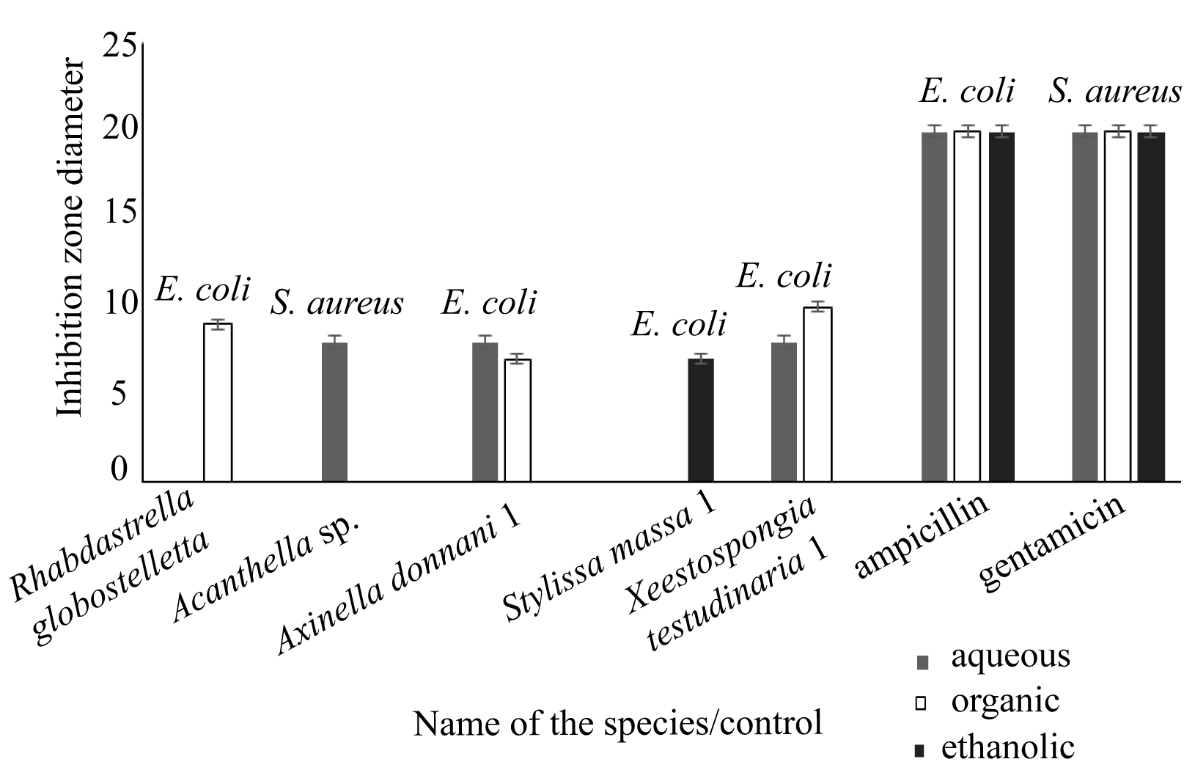


**S3 Fig. Agar disc diffusion assay.** The specimens that exhibited antibacterial activity against *E. coli* and/or *S. aureus* in the agar disc diffusion assay are shown. Ampicillin and gentamicin were used as the positive controls. The data are represented as the mean diameter of the inhibition zone ± standard deviation.


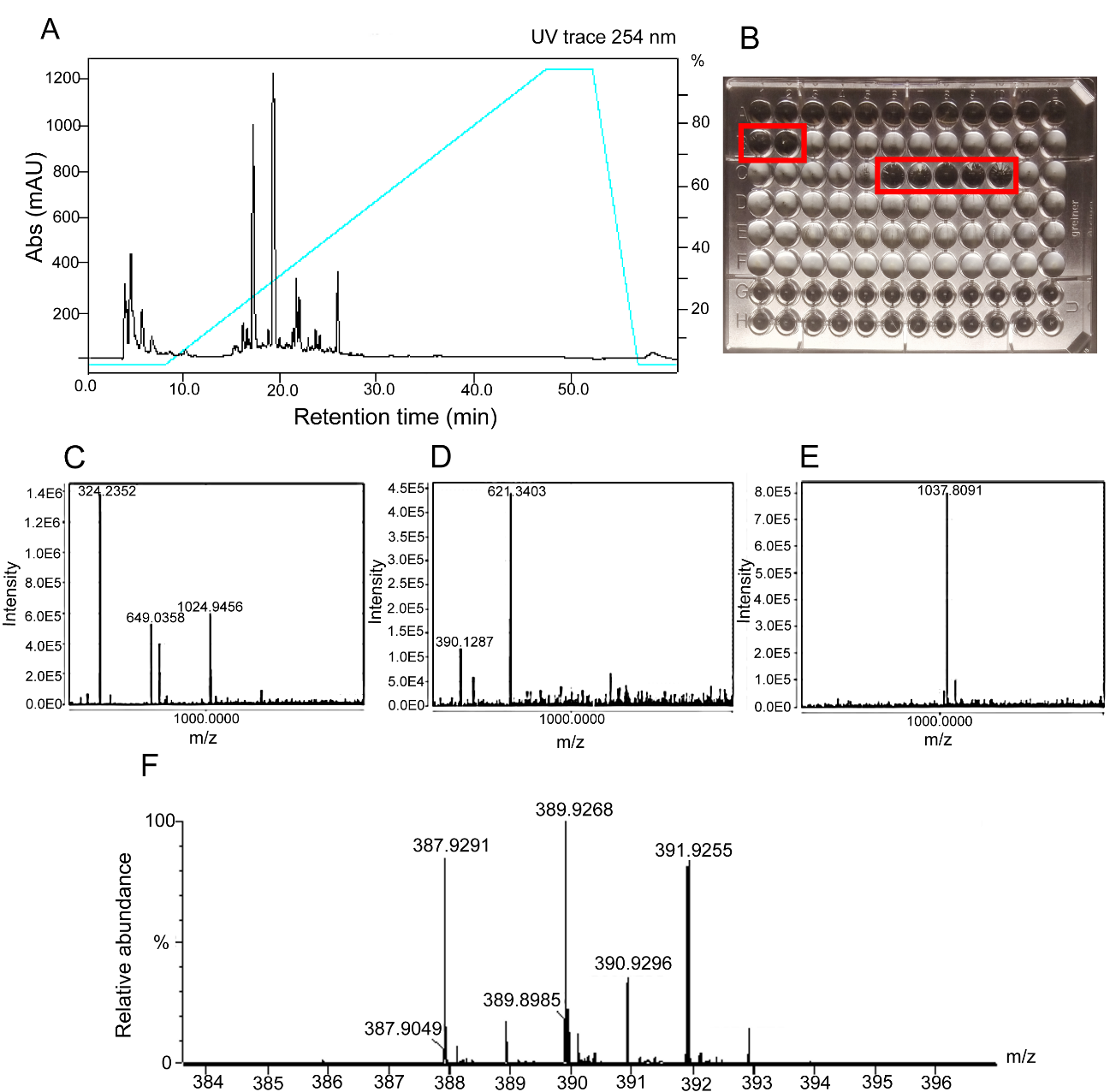


**S4 Fig. Isolation and identification of bromopyrrole alkaloids from *Stylissa massa*** 2**.** (A) The RP-HPLC chromatogram of the ethanolic extract of *Stylissa massa* 2 detected at 254 nm, (B) Microfractionated ethanolic extract of *Stylissa massa* 2 showing a total growth inhibition for bioactive wells 1, 2 and 15 to 19 in the microdilution assay, (C-E) the mass spectra for the bioactive fractions showing the most abundant masses, (F) the isotopic peak ratio of 1:2:1 for hymenin

**S1 Table. Extracts with no antibacterial activity in the disc diffusion and microdilution assay**

| No | Name of the specimen | Aqueous extracts (60% MeOH) | | | Organic extracts (DCM: MeOH 9:1) | | | Ethanolic extracts (98% ethanol) | | |
| --- | --- | --- | --- | --- | --- | --- | --- | --- | --- | --- |
|  |  | Disc diffusion assay | Microdilution assay | | Disc diffusion assay | Microdilution assay | | Disc diffusion assay | Microdilution assay | |
|  |  |  | Tested against  *S. aureus* | Tested against  *E. coli* |  | Tested against  *S. aureus* | Tested against  *E. coli* |  | Tested against  *S. aureus* | Tested against  *E. coli* |
| 1 | *Manihinea* sp. |  |  |  | X | X | X |  |  |  |
| 2 | *Petrosia (Petrosia)* sp. 1 | X | X | X |  |  |  |  |  |  |
| 3 | *Spongosorites* sp. | X | X | X |  |  |  |  |  |  |
| 4 | *Amorphinopsis foetida* |  |  |  | X | X | X |  |  |  |
| 5 | *Stylissa massa* 2 | X | X | X | X | X | X | X | X | X |
| 6 | *Plakinastrella ceylonica* |  |  |  |  |  |  | X | X | X |
| 7 | *Erylus* sp. |  |  |  | X | X | X |  |  |  |
| 8 | *Topsentia* sp. | X | X | X |  |  |  |  |  |  |
| 9 | *Haliclona (Reniera)* sp.1 | X | X | X |  |  |  | X | X | X |
| 10 | *Paratetilla bacca* | X | X | X |  |  |  | X | X | X |
| 11 | *Stellitethya* sp. |  |  |  |  |  |  | X | X | X |
| 12 | *Ircinia* sp. | X | X | X | X | X | X | X | X | X |
| 13 | *Cliona* sp. | X | X | X |  |  |  | X | X | X |
| 14 | *Haliclona* sp. |  |  |  |  |  |  | X | X | X |
| 15 | *Haliclona (Reniera)* sp.2 |  |  |  |  |  |  | X | X | X |
| 16 | *Haliclona (Reniera)* sp.3 |  |  |  |  |  |  | X | X | X |
| 17 | *Spheciospongia inconstans* 1 |  |  |  |  |  |  | X | X | X |
| 18 | *Spheciospongia inconstans* 2 |  |  |  | X | X | X | X | X | X |
| 19 | *Spheciospongia inconstans* 3 |  |  |  |  |  |  | X | X | X |

X indicates specimens that were screened in the relevant assay but gave no bioactivity.

Blank columns represent extracts that were not tested in the relevant assay due to inadequate material.

**S2 Table. Systematic literature review.** Antibacterial and cytotoxic compounds reported in previous studies that are isolated from the species described in the present study. WPD: World Porifera Database, <https://www.marinespecies.org/porifera>.

| **Source organism** | **Name of the compound and the molecular formula** | **Monoisotopic mass/Da** | **Bioactivity** | **Monoisotopic masses found in the present study in Da**  **[M + H]^+^** | **Reference** |
| --- | --- | --- | --- | --- | --- |
| *Aspergillus similanensis* KUFA 0013 (fungus) isolated from the sponge *Rhabderemia* sp. | chevalone E  C_26_H_38_O_4_ | 415.6  [M + H]^+^ | synergism with the antibiotic oxacillin against methicillin-resistant *Staphylococcus aureus* (MRSA) | 1043.44  885.58  1792.69  885.83 | (1) |
| *Strongylophora* sp*.*  (= *Petrosia (Strongylophora)* in the WPD) | strongylophorine-2  C_26_H_34_O_2_ | 410 | Antimicrobial activity (M. lueus, S. typhi, C. cucumerinum) | 1039.77, 353.44, 1791.75, 885.98, 411.71, 427.76, 493.08 | (2) |
|  | strongylophorine-3  C_26_H_36_O_2_ | 412 |  |  |  |
| *Strongylophora* durissima  (= *Petrosia (Strongylophora)* durissima in the WPD) | strongylophorine-6  C_26_H_34_O_5_ | 426.2415 | antimicrobial activity (*B. subtilis*, *S. aureus*) |  | (3) |
|  | strongylophorine-7  C_26_H_34_O_5_ | 426.2398 |  |  |  |
| *Erylus nobilis* | Eryloside G  C_50_H_81_NO_17_ | 990.5401  [M + Na]^+^ | mild cytotoxic effect against the human leukemia cell-line K562 with LC_50_ values of 22.1, 24.8, 17.9, and 21.8 µg/mL respectively | 1065.68  1095.69  712.42 | (4) |
|  | Eryloside H  C_49_H_79_NO_16_ | 960.5320 [M+Na]^+^ |  |  |  |
|  | Eryloside I  C_51_H_83_NO_17_ | 1004.5545 [M+Na]^+^ |  |  |  |
|  | Eryloside J  C_50_H_81_NO_16_ | 974.5436 [M+Na]^+^ |  |  |  |
|  | Heterocornol B  C_14_H_18_O_4_ | 251.29  [M + H]^+^ |  |  |  |
|  | Heterocornol C  C_12_H_14_O_3_ | - |  |  |  |
|  | Heterocornol F  C_17_H_24_O_4_ | 315.1651 [M+Na]^+^ |  |  |  |
|  | Heterocornol G  C_17_H_22_O_3_ | 273.1504  [M-H]^-^ |  |  |  |
|  | Heterocornol H | - |  |  |  |
|  | Vaccinol G  C_17_H_22_O_4_ | 290.2 |  |  |  |
|  | Agropyrenol  C_12_H_14_O_4_ | 245.0801 [M+Na]+ |  |  |  |
| *Aulospongus gardineri* | No reported antibacterial compounds from *Aulospongus gardineri* | | | 735.15, 885.35, 1791.36 |  |
| *Manihinea* sp. | Aurantoside C  C_37_H_46_Cl_2_O_2_N_15_ | 829.6216  [M + H]^+^ | cytotoxic activities in Triple negative breast cancer cells | 296.03  304.03 | (5) |
| *Halichondria*sp. | halichonadin A  C_31_H_52_N_2_O | 468.4079  [M + H]^+^ | antibacterial activity against Micrococcus luteus | 1387.75  1039.84  1086.59  328.59 | (6) |
|  | halichonadin B  C_17_H_29_NO_2_ | 279.2203  [M + H]^+^ |  |  |  |
|  | halichonadin C  C_16_H_25_N | 231.1987  [M + H]^+^ |  |  |  |
|  | halichonadin D  C_15_H_27_N | 221.2135  [M + H]^+^ |  |  |  |
| *Acanthella* sp. | Kalihinol A  C_22_H_33_ClN_2_O_2_ |  | cytotoxic activity against HCT-116 | 373.31  269.25  415.55  356.2  848.78 | (7) |
|  | Kalihinol E  C_22_H_33_ClN_2_O_2_ |  |  |  |  |
|  | Kalihinol O  C_22_H_33_ClN_2_O_2_SNa | 447.1850 [M+Na]^+^ |  |  |  |
|  | Kalihinol P  C_22_H_33_ClN_2_O_2_SNa | 447.1846 [M+Na]^+^ |  |  |  |
|  | Kalihinol Q  C_22_H_33_ClN_2_O_2_SNa | 447.1847 [M+Na]^+^ |  |  |  |
|  | Kalihinol R  C_22_H_33_ClN_2_O_2_S_2_Na | 479.1571 [M+Na]^+^ |  |  |  |
|  | 10-epi-kalihinol I  C_22_H_33_ClN_2_O_2_S | - |  |  |  |
|  | 10-epi-kalihinol X | - |  |  |  |
| *Axinella donnani* | Cerebroside | 208 | Antibaacterial activity against *Aeromonas hydrophila* subsp. *Salmonicida* A449 and *Erythrobacter litoralis, Alcanivorax* sp. and *Alcanivorax borkumensis* | 376.26, 200.23,  230.24, 561.49 | (8) |
|  | Lectin |  | Antibacterial activity against *E. coli,*  *K. pneumoniae,*  *P. aeruginosa,*  *S. aureus* |  | (9) |
| *Topsentia* sp. | Topsentiasterol sulfate A  C_30_H_43_O_16_S_3_Na_3_ | 801.1488  (M-Na)^-^ | antibacterial against  *P. aeruginosa* and *E. coli* | 497.86 | (10) |
|  | Topsentiasterol sulfate B  C_30_H_43_O_16_S_3_Na_3_ | - |  |  |  |
|  | Topsentiasterol sulfate C  C_30_H_43_O_15_S_3_Na_3_ | - |  |  |  |
|  | Topsentiasterol sulfate D  C_30_H_43_O_14_S_3_Na_3_ | - |  |  |  |
|  | Topsentiasterol sulfate E  C_31_H_49_O_13_S_3_Na_3_ | 363.12124  ([M − 3Na + H]^−2^ |  |  |  |
| *Topsentia pachastrelloides* | Cis-3,4-dihyrohyrohamacanthin B | - | MRSA PK inhibitory activity |  | (11) |
| *Siphonochalina siphonella* (= *Callyspongia (Callyspongia) siphonella* in WPD) | Siphonodiol  C_23_H_24_O_2_ | 332.1760  [M+H]^+^ | Antibacterial activity against *S. aureus* and *Streptococcus pyogenes* | 421.42  885.81  281.55  309.63 | (12) |
|  | Neviotine A  C_30_H_50_O_6_ | 506.7143  [M+H]^+^ | Antibacterial activity against *S. aureus*, *B. subtilis* and *E. coli* |  |  |
|  | Sipholenol L  C_30_H_52_O_4_ | 476.4865 | Antibacterial activity against *S. aureus* and *B. subtilis* |  |  |
|  | Sipholenone A  C_30_H_50_O_4_ | 474.37  [M+H]^+^ | Antibacterial activity against *S. aureus,* *B. subtilis* and *E. coli* |  |  |
| *Callyspongia siphonella* | 5-bromotrisindoline  C_24_H_16_ON_3_Br | 442.0554  [M + H]^+^ | Antibacterial activity against *S. aureus,* *B. subtilis* with inhibition zones of 17.5 mm and 18 mm respectively |  | (13) |
|  | 6-bromotrisindoline  C_24_H_16_ON_3_Br | 442.0554  [M + H]^+^ | Antibacterial activity against *S. aureus,* *B. subtilis* with inhibition zones of 15 mm and 16.4 mm respectively |  |  |
| *Aciculites orientalis* | Aciculitin A  C_61_H_86_N_14_O_21_ | - | Inhibited the growth of *C. albicans* and showed cytotoxicity against HCT-116 cell line | 222.20  295.52  595.79  355.39 | (14) |
|  | Aciculitin B  C_62_H_88_N_14_O_21_ | - |  |  |  |
|  | Aciculitin C  C_63_H_90_N_14_O_21_ | - |  |  |  |
| *Agelas ceylonica* | methyl ester of hanishin |  | showed mild antibacterial activity against *B. subtilis* | 401.85,  470.92 | (15) |
| *Agelas* sp. | Sceptrin  [C_22_H_24_Br_2_N_10_O_2_](https://pubchem.ncbi.nlm.nih.gov/#query=C22H24Br2N10O2) | 621.3  [M + H]^+^ | Antimicrobial properties |  | (16) |
|  | Hexazosceptrin  [C_22_H_24_Br_2_N_10_O_3_](https://pubchem.ncbi.nlm.nih.gov/#query=C22H24Br2N10O3) | 633.0328  [M + H]^+^ |  |  | (17) |
|  | Agelamadin A  C_23_H_26_N_10_O_3_Br_4_ | 804.88553  [M + H]^+^ |  |  | (18) |
|  | Agelamadin B  C_22_H_24_N_10_O_3_Br_4_ | 790.86975  [M + H]^+^ |  |  |  |
|  | Nagelamide A  C_22_H_22_O_2_N_10_Br_4_ | 774.8735  (M +H)+ |  |  |  |
|  | Nagelamide B  C_22_H_22_O_3_N_10_Br_4_ | 789.8651  [M + H]^+^ |  |  |  |
|  | Nagelamide C  C_22_H_20_O_2_N_10_Br_4_ | 772.8596  (M + H)+ |  |  |  |
|  | Nagelamide D  C_22_H_24_O_2_N_10_Br_4_ | 776.8735  [M + H]^+^ |  |  |  |
|  | Nagelamide E  C_22_H_24_N_10_O_2_Br_2_ | 619.0517  [M + H]^+^ |  |  |  |
|  | Nagelamide F  C_22_H_23_N_10_O_2_Br_3_ | 696.9609  [M + H]^+^ |  |  |  |
|  | Nagelamide G  C_22_H_22_N_10_O_2_Br | 774.8726  [M + H]^+^ |  |  |  |
|  | Nagelamide H  C_24_H_24_O_5_N_11_SBr_4_ | 897.8408  (M + 2 - H)^-^ |  |  |  |
|  | Nagelamide O  C_22_H_24_Br_3_ClN_10_O_4_ | 764.9315  (M + H)^+^ |  |  |  |
| *Amorphinopsis* sp. | No reported antibacterial compounds from *Amorphinopsis* sp. | | | 1931.70, 393.61, 1792.64 |  |
| *Stylissa massa* | Sceptrin  [C_22_H_24_Br_2_N_10_O_2_](https://pubchem.ncbi.nlm.nih.gov/#query=C22H24Br2N10O2) | 621.3  [M + H]^+^ | inhibitory effects on cell motility in human cervical cancer HeLa cell line | 1037.68  324.00  202.08  799.46  374.24  173.95 | (19) |
|  | Spongiacidin C  C_11_H_10_N_4_O_3_ | 246.0729  [M]^+^ | USP7 inhibitor |  | (20) |
|  | Stylissatins B  C_38_H_51_N_9_O_7_ | 746.4003  [M + H]^+^ | inhibitory effects against a panel of human tumor cell lines including HCT-116, HepG2, BGC-823, NCI-H1650, A2780, and MCF7 |  | (21) |
|  | Dibromoisophakellin  C_11_H_11_N_5_OBr_2_ | - | significantly reduced the biofilm formation of the bacterium *E. coli* |  | (22) |
|  | Dibromophakellin  C_11_H_11_N_5_OBr_2_ | - |  |  |  |
| *Xestospongia testudinaria* | Prodigiosin  C_20_H_25_N_3_O | 324.3  [M + H]^+^ | Antibacterial properties | 421.40  376.28 | (23) |
|  | 18,18-dibromo- (9E)-octadeca-9,17-diene-5,7-diynoic acid | - | cytotoxic properties against the human cervical cancer cell line (HeLa) |  | (24) |
|  | 18-bromooctadeca-(9E,17E)-diene-7,15-diynoic acid  C_18_H_22_BrO_2_ | 351  [M-H]^-^ | cytotoxic properties against human medulloblastoma cancer cell line (Daoy) |  |  |
|  | 16-bromo (7E,11E,l5E)hexadeca-7,11,l5-triene-5,13-diynoic acid | - | cytotoxic properties against human medulloblastoma cancer cell line (Daoy) |  |  |
| *Spheciospongia vesparia* | Svl-1 | 12146.7 | Antibacterial activity against *P. aeruginosa* and *E. coli* | 202.09,  389.94,  242.98,  446.21  204.09  979.64  4913.20  4897.24  324.01  230.24  385.13 | (25) |
|  | Svl-2 | 12209.3 |  |  |  |
| *Micrococcus* sp. EG45 cultivated from the Red Sea sponge *Spheciospongia vagabunda* | microluside A  C_45_H_47_O_23_ | [M+1]+  955.2511 | antibacterial potential against *Enterococcus faecalis* JH212 (MIC= 10 µM) and *Staphylococcus aureus* NCTC 8325 (MIC= 13 µM) |  | (26) |
| *Tedania (Tedania)* sp. | No reported antibacterial compounds from *Tedania (Tedania)* sp. | | | 137.08 |  |

**References**

1. Prompanya C, Dethoup T, Bessa LJ, Pinto MMM, Gales L, Costa PM, et al. New Isocoumarin Derivatives and Meroterpenoids from the Marine Sponge-Associated Fungus *Aspergillus similanensis* sp. nov. KUFA 0013. Marine Drugs. 2014;12(10):5160-73.

2. Balbin-Oliveros M, Edrada RA, Proksch P, Wray V, Witte L, Van Soest RW. A new meroditerpenoid dimer from an undescribed Philippine marine sponge of the genus *Strongylophora*. Journal of natural products. 1998;61(7):948-52.

3. Salva J, Faulkner DJ. Metabolites of the sponge *Strongylophora durissima* from Maricaban Island, Philippines. The Journal of Organic Chemistry. 1990;55(6):1941-3.

4. Lee H-S, Rho J-R, Shin J. Bioactive metabolites from sponges-new triterpenoid saponins from the sponge *Erylus nobilis*. Fisheries science. 2002;68(sup2):1597-600.

5. Shrestha S, Sorolla A, Fromont J, Blancafort P, Flematti GR. Aurantoside C Targets and Induces Apoptosis in Triple Negative Breast Cancer Cells. Marine Drugs. 2018;16(10):361.

6. Ishiyama H, Hashimoto A, Fromont J, Hoshino Y, Mikami Y, Kobayashi Ji. Halichonadins A–D, new sesquiterpenoids from a sponge *Halichondria* sp. Tetrahedron. 2005;61(5):1101-5.

7. Xu Y, Li N, Jiao W-H, Wang R-P, Peng Y, Qi S-H, et al. Antifouling and cytotoxic constituents from the South China Sea sponge *Acanthella cavernosa*. Tetrahedron. 2012;68(13):2876-83.

8. Majik MS, Shirodkar D, Rodrigues C, D’Souza L, Tilvi S. Evaluation of single and joint effect of metabolites isolated from marine sponges, *Fasciospongia cavernosa* and *Axinella donnani* on antimicrobial properties. Bioorganic & Medicinal Chemistry Letters. 2014;24(13):2863-6.

9. Sadanandan R, Rauf AA. Antibacterial activity of a lectin isolated from marine sponge *Axinella donnani*. J Aquat Biol Fish. 2018;6:159-64.

10. Fusetani N, Takahashi M, Matsunaga S. Topsentiasterol sulfates, antimicrobial sterol sulfates possessing novel side chains, from a marine sponge, *Topsentia* sp. Tetrahedron. 1994;50(26):7765-70.

11. Zoraghi R, Worrall L, See RH, Strangman W, Popplewell WL, Gong H, et al. Methicillin-resistant *Staphylococcus aureus* (MRSA) pyruvate kinase as a target for bis-indole alkaloids with antibacterial activities. Journal of Biological Chemistry. 2011;286(52):44716-25.

12. Al-Massarani SM, El-Gamal AA, Al-Said MS, Al-Lihaibi SS, Basoudan OA. In vitro cytotoxic, antibacterial and antiviral activities of triterpenes from the Red Sea sponge, *Siphonochalina siphonella*. Tropical Journal of Pharmaceutical Research. 2015;14(1):33-40.

13. El-Hawary SS, Sayed AM, Mohammed R, Hassan HM, Rateb ME, Amin E, et al. Bioactive brominated oxindole alkaloids from the Red Sea sponge *Callyspongia siphonella*. Marine drugs. 2019;17(8):465.

14. Bewley CA, He H, Williams DH, Faulkner DJ. Aciculitins A−C:  Cytotoxic and Antifungal Cyclic Peptides from the Lithistid Sponge *Aciculites orientalis*. Journal of the American Chemical Society. 1996;118(18):4314-21.

15. Srinivasa Reddy N, Venkateswarlu Y. S-(+)-methyl ester of hanishin from the marine sponge *Agelas ceylonica*. Biochemical Systematics and Ecology. 2000;28(10):1035-7.

16. Walker RP, Faulkner DJ, Van Engen D, Clardy J. Sceptrin, an antimicrobial agent from the sponge *Agelas sceptrum*. Journal of the American Chemical Society. 1981;103(22):6772-3.

17. Sun Y-T, Lin B, Li S-G, Liu M, Zhou Y-J, Xu Y, et al. New bromopyrrole alkaloids from the marine sponge *Agelas* sp. Tetrahedron. 2017;73(19):2786-92.

18. Zhang H, Dong M, Chen J, Wang H, Tenney K, Crews P. Bioactive secondary metabolites from the marine sponge genus *Agelas*. Marine drugs. 2017;15(11):351.

19. Cipres A, O’Malley DP, Li K, Finlay D, Baran PS, Vuori K. Sceptrin, a Marine Natural Compound, Inhibits Cell Motility in a Variety of Cancer Cell Lines. ACS Chem Biol. 2010;5(2):195-202.

20. Sun J, Cheng W, de Voogd NJ, Proksch P, Lin W. Stylissatins B–D, cycloheptapeptides from the marine sponge *Stylissa massa*. Tetrahedron Letters. 2016;57(38):4288-92.

21. Yamaguchi M, Miyazaki M, Kodrasov MP, Rotinsulu H, Losung F, Mangindaan RE, et al. Spongiacidin C, a pyrrole alkaloid from the marine sponge *Stylissa massa*, functions as a USP7 inhibitor. Bioorganic & medicinal chemistry letters. 2013;23(13):3884-6.

22. Sun J, Wu J, An B, Voogd NJd, Cheng W, Lin W. Bromopyrrole alkaloids with the inhibitory effects against the biofilm formation of Gram negative bacteria. Marine drugs. 2018;16(1):9.

23. Ibrahim D, Nazari TF, Kassim J, Lim S-H. Prodigiosin-an antibacterial red pigment produced by *Serratia marcescens* IBRL USM 84 associated with a marine sponge *Xestospongia testudinaria*. Journal of Applied Pharmaceutical Science. 2014;4(10):001-6.

24. El-Gamal AA, Al-Massarani SM, Shaala LA, Alahdald AM, Al-Said MS, Ashour AE, et al. Cytotoxic compounds from the Saudi Red Sea sponge *Xestospongia testudinaria*. Marine drugs. 2016;14(5):82.

25. Fenton B, Espinosa RA, Contreras EV, Lozano BA, Sánchez NS, Hernández EG, et al. Purification and characterization of structural and functional properties of two lectins from a marine sponge *Spheciospongia vesparia*. 2013.

26. Eltamany EE, Abdelmohsen UR, Ibrahim AK, Hassanean HA, Hentschel U, Ahmed SA. New antibacterial xanthone from the marine sponge-derived *Micrococcus* sp. EG45. Bioorganic & medicinal chemistry letters. 2014;24(21):4939-42.
