# Supplementary figures and images for: Screening for antibacterial and cytotoxic activities of Sri Lankan marine sponges through microfractionation: Isolation of bromopyrrole alkaloids from Stylissa massa
Source: PLoS One. 2024 Jan 8;19(1):e0296404. doi: 10.1371/journal.pone.0296404 (PMC10773956; doi:10.1371/journal.pone.0296404)

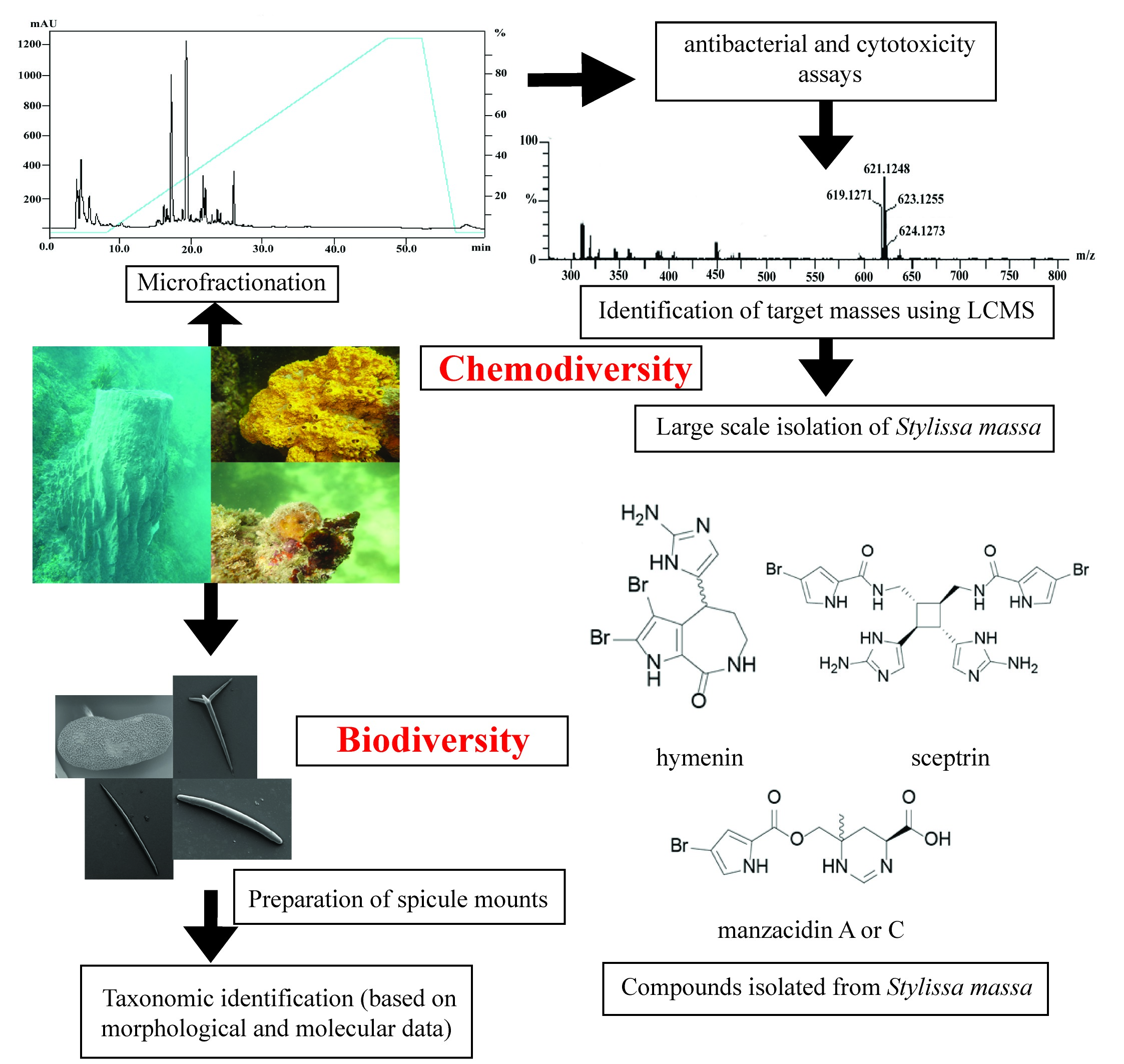

Supplement: S1 Fig — (TIF) [file pone.0296404.s002.tif]
