# Supplementary material for: Screening for antibacterial and cytotoxic activities of Sri Lankan marine sponges through microfractionation: Isolation of bromopyrrole alkaloids from Stylissa massa
Source: PLoS One. 2024 Jan 8;19(1):e0296404. doi: 10.1371/journal.pone.0296404 (PMC10773956; doi:10.1371/journal.pone.0296404)
Supplement: Attachment — Submitted filename: Response to reviewers.pdf [file pone.0296404.s005.pdf]

Awatif Abid Al-Judaibi, PhD
Academic Editor
PLOS ONE

December 05th, Uppsala

Dear Dr. Awatif Abid Al-Judaibi,

We appreciate your time and the valuable feedback provided by the editor and Reviewers on our manuscript "Screening for antibacterial and cytotoxic activities of Sri Lankan marine sponges through microfractionation: Isolation of bromopyrrole alkaloids from *Stylissa massa*" (PONE-D-23-15586R1). We have thoroughly reviewed the comments and have addressed each point raised by the editor and Reviewers as given below.

We appreciate the opportunity to improve our manuscript and looking forward to a favourable response.

Yours sincerely,

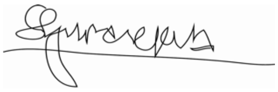

………………………………..

Sunithi Gunasekera (PhD)
Associate Professor
Department of Pharmaceutical Biosciences
Uppsala University

Editor's comments:

1- **Please review your reference list to ensure that it is complete and correct. If you have cited papers that have been retracted, please include the rationale for doing so in the manuscript text, or remove these references and replace them with relevant current references. Any changes to the reference list should be mentioned in the rebuttal letter that accompanies your revised manuscript. If you need to cite a retracted article, indicate the article's retracted status in the References list and also include a citation and full reference for the retraction notice:**
We have carefully reviewed the reference list and confirm that it is complete and correct. None of the cited papers have been retracted. No changes were made to the reference list during revising.

Reviewer 1's comments:

1- **Line 408 and 423- Change "literature values" to "Previously published values":**
Corrected

2- **Line 457- Change A, orientalis to A. orientalis:**
Corrected

3- **Combine lines 514 to 517 to a single sentence:**
Corrected

Reviewer 3's comments:

1- **It is recommended to carry out future in vivo animal studies to confirm the in vitro enzyme assay:**

We would appreciate your suggestion and look forward to conducting *in vivo* animal studies as a future plan.
