# Supplementary material for: Screening for antibacterial and cytotoxic activities of Sri Lankan marine sponges through microfractionation: Isolation of bromopyrrole alkaloids from Stylissa massa
Source: PLoS One. 2024 Jan 8;19(1):e0296404. doi: 10.1371/journal.pone.0296404 (PMC10773956; doi:10.1371/journal.pone.0296404)
Supplement: Attachment — Submitted filename: Response.docx [file pone.0296404.s004.docx]

Line 408 and 423- Change “literature values” to “Previously published values”.

Line 456- Change A, orientalis to A. orientalis

Combine lines 513 to 516 to a single sentence.
